# Construct Validity and Reliability of the SARA Gait and Posture Sub-scale in Early Onset Ataxia

**DOI:** 10.3389/fnhum.2017.00605

**Published:** 2017-12-13

**Authors:** Tjitske F. Lawerman, Rick Brandsma, Renate J. Verbeek, Johannes H. van der Hoeven, Roelineke J. Lunsing, Hubertus P. H. Kremer, Deborah A. Sival

**Affiliations:** Departments of Pediatrics and Neurology, Beatrix Children’s Hospital, University Medical Center Groningen, Groningen, Netherlands

**Keywords:** early onset ataxia, SARA, gait, validity, myopathy, muscle weakness, coordination, balance

## Abstract

**Aim:** In children, gait and posture assessment provides a crucial marker for the early characterization, surveillance and treatment evaluation of early onset ataxia (EOA). For reliable data entry of studies targeting at gait and posture improvement, uniform quantitative biomarkers are necessary. Until now, the pediatric test construct of gait and posture scores of the Scale for Assessment and Rating of Ataxia sub-scale (SARA) is still unclear. In the present study, we aimed to validate the construct validity and reliability of the pediatric (SARA_GAIT/POSTURE_) sub-scale.

**Methods:** We included 28 EOA patients [15.5 (6–34) years; median (range)]. For inter-observer reliability, we determined the ICC on EOA SARA_GAIT/POSTURE_ sub-scores by three independent pediatric neurologists. For convergent validity, we associated SARA_GAIT/POSTURE_ sub-scores with: (1) Ataxic gait Severity Measurement by Klockgether (ASMK; dynamic balance), (2) Pediatric Balance Scale (PBS; static balance), (3) Gross Motor Function Classification Scale -extended and revised version (GMFCS-E&R), (4) SARA-kinetic scores (SARA_KINETIC_; kinetic function of the upper *and* lower limbs), (5) Archimedes Spiral (AS; kinetic function of the upper limbs), and (6) total SARA scores (SARA_TOTAL_; i.e., summed SARA_GAIT/POSTURE_, SARA_KINETIC_, and SARA_SPEECH_ sub-scores). For discriminant validity, we investigated whether EOA co-morbidity factors (myopathy and myoclonus) could influence SARA_GAIT/POSTURE_ sub-scores.

**Results:** The inter-observer agreement (ICC) on EOA SARA_GAIT/POSTURE_ sub-scores was high (0.97). SARA_GAIT/POSTURE_ was strongly correlated with the other ataxia and functional scales [ASMK (*r*_s_ = -0.819; *p* < 0.001); PBS (*r*_s_ = -0.943; *p* < 0.001); GMFCS-E&R (*r*_s_ = -0.862; *p* < 0.001); SARA_KINETIC_ (*r*_s_ = 0.726; *p* < 0.001); AS (*r*_s_ = 0.609; *p* = 0.002); and SARA_TOTAL_ (*r*_s_ = 0.935; *p* < 0.001)]. Comorbid myopathy influenced SARA_GAIT/POSTURE_ scores by concurrent muscle weakness, whereas comorbid myoclonus predominantly influenced SARA_KINETIC_ scores.

**Conclusion:** In young EOA patients, separate SARA_GAIT/POSTURE_ parameters reveal a good inter-observer agreement and convergent validity, implicating the reliability of the scale. In perspective of incomplete discriminant validity, it is advisable to interpret SARA_GAIT/POSTURE_ scores for comorbid muscle weakness.

## Introduction

Pediatric ataxic gait and posture- assessment provides an important instrument to identify children and young adults with indisputable EOA (Brandsma et al., 2016a; Lawerman et al., 2016). The availability of validated gait and posture- biomarkers in children is also important for the entry of high quality data in international EOA databases (Durr, 2015; Brandsma et al., 2016a; Lawerman et al., 2016) and also for the evaluation of treatment (Romano et al., 2015), especially when the training of core-muscles is involved (such as by exergame-training) (van Diest et al., 2016; Schatton et al., 2017). In young, often disabled, EOA patients with limited concentration and physical endurance, optimally applicable gait and posture biomarkers are characterized as: non-invasive, quick and easy, compatible with adult parameters, reliable and also associated with a good construct validity (Schmidt and Embretson, 2003; Saute et al., 2012). Until now, insight in the validity of clinically available gait and posture- biomarkers is incomplete. The SARA is described as a reliable, quickly assessable, and non-invasive rating scale for patients with ataxia (Schmitz-Hubsch et al., 2006). SARA scores consist of summed: gait and posture- (SARA_GAIT/POSTURE_ measuring gait, stance, sitting performances), kinetics (SARA_KINETIC_) and speech (SARA_SPEECH_) sub-scores (Schmitz-Hubsch et al., 2006). In EOA, we aimed to investigate the construct validity of the pediatric SARA_GAIT/POSTURE_ sub-scale scores.

For the investigation of the EOA SARA_GAIT/POSTURE_ construct validity, it is important to realize two points. First, it is important to realize that the SARA was originally designed and validated as a complete, total score in the domains of gait/posture, kinetics, and speech (Schmitz-Hubsch et al., 2006). However, under the assumption that the SARA sub-scale scores SARA_GAIT/POSTURE_ and SARA_KINETIC_ measure cerebellar functioning in different domains (i.e., vermis and anterior lobe and cerebellar hemispheres, respectively), we hypothesized that the SARA_GAIT/POSTURE_ sub-scale could be separately validated. Second, it is important to realize that the SARA was originally designed and validated in adult patients with AOA (Schmitz-Hubsch et al., 2006). However, due to the short clinical assessment time and good score reproducibility, the scale was soon applied in children too (Brandsma et al., 2014a, 2016b; Hartley et al., 2015; Reetz et al., 2015). Before SARA scores can be analogously interpreted in AOA and EOA patients, it is thus important to take the effect of potential group differences into account. In comparison with the AOA patient group, EOA patients may reveal a large variety of disorders, with a heterogeneous phenotypic presentation and co-morbidity (such as myopathy and/or myoclonus). This explains why SARA score characteristics can differ between AOA and EOA patient groups (Sival and Brunt, 2009; Sival et al., 2011; Brandsma et al., 2014a, 2016b). For instance, in AOA patients, total SARA scores relate with ataxia as one single factor [i.e., ‘ataxia’ (Schmitz-Hubsch et al., 2006)]. This is contrasted by total SARA scores in EOA patients, which are also attributed to: (1) pediatric age (i.e., cerebellar maturation; Largo et al., 2003; Sival and Brunt, 2009; Brandsma et al., 2014a), (2) comorbid muscle weakness [in FA (Sival et al., 2011)], and (3) comorbid movement disorders (Brandsma et al., 2016b).

In children and young adults with EOA, we thus aimed to investigate the construct validity of the SARA_GAIT/POSTURE_ sub-scale. Under the premise that parameters for SARA_GAIT/POSTURE_ would depend on the integrated cerebellar processing of visual, vestibular, and sensory signals of the limbs and trunk (Sival, 2012; Delabasita et al., 2016; Takakusaki, 2017), SARA_GAIT/POSTURE_ sub-scales would be expected to correlate with biomarkers for dynamic and passive balance, such as: the scale for ASMK [dynamic balance (Klockgether et al., 1998)] and the PBS (static balance; Franjoine et al., 2010). Additionally, we reasoned that clinically meaningful and effective SARA_GAIT/POSTURE_ sub-scores would relate with a validated, age-related classification system for functional motility in children, such as the GMFCS (Palisano et al., 1997) – the extended and revised version (E&R; Palisano et al., 2008), which is originally designed for children with cerebral palsy. Furthermore, accurate kinematics for SARA_GAITPOSTURE_ performances would also correlate with biomarkers for kinetic-limb function, such as: SARA_KINETIC_ (upper and lower limbs) and AS [upper limb kinetic scores (Trouillas et al., 1997)]. Finally, effective EOA SARA_GAIT/POSTURE_ scores would be expected to correlate with SARA_TOTAL_. Strong and significant correlations would underpin a good convergent validity of SARA_GAIT/POSTURE_ sub-scale scores. Absent influence by EOA co-morbidity factors (such as muscle weakness and/or myoclonus) on the scores would underpin sufficient discriminant validity of the SARA_GAIT/POSTURE_ sub-scale.

In the present study, we thus aimed to elucidate the construct validity and reliability of EOA SARA_GAIT/POSTURE_ sub-scale scores in children and young adults.

## Materials and Methods

The Medical Ethical Committee of the University Medical Center Groningen (UMCG), Netherlands, approved the study (METc 2011/165). According to the Dutch medical ethical law, both parents and children older than 12 years of age provided written informed consent. Children younger than 12 years of age provided assent. All subjects gave written informed consent in accordance with the Declaration of Helsinki. The protocol was approved by the ‘The Medical Ethical Committee of the University Medical Center Groningen (UMCG), Netherlands’. In the absence of preceding pediatric data for a power calculation, we performed a prospective, explorative study.

### Patients

Over a 5 year period (2011–2016), we have collected a complete cohort of EOA children that visited the pediatric neurology ward at UMCG (Brandsma et al., 2016b). From this cohort, we included patients that fulfilled the criteria for “distinct ataxia,” characterized by: EOA (initiation of ataxia before the 25th year of life) and unanimous recognition of ataxia as the main movement disorder by three independent pediatric neurologists and/or unanimous recognition of ataxia as part of the movement disorder by three independent pediatric neurologists *and* confirmation of the ataxic phenotype by the OMIM database^1^. Patients were excluded when they were unable to understand the required motor function tasks for the present study.

We included 28 EOA patients [median age 15.5 (range: 6–34) years]. The response rate was 100%. In 24/28 (86%) patients, ataxia was independently recognized as the main movement disorder by all three pediatric neurologists. The other 4 of 28 (14%) patients were included on basis of unanimous phenotypic ataxia recognition (primary or secondary features) *and* diagnostic confirmation that ataxia is involved according to the OMIM database^1^. Underlying metabolic or genetic diagnoses (*n* = 24/28) included: FA (*n* = 8), GOSR2-mutation (*n* = 4), ataxia with vitamin E deficiency (AVED; *n* = 2), CACNA1A-mutation (*n* = 2), Ataxia Telangiectasia (*n* = 1), Joubert syndrome type 23 (*n* = 1), Kearns Sayre syndrome (KSS; *n* = 1), MHBD-deficiency (*n* = 1), NARP-mutation (*n* = 1), Niemann–Pick type C (*n* = 1), Poretti Bolthauser syndrome (*n* = 1), and SCA5 (*n* = 1). The remaining four patients remained undiagnosed, despite whole exome sequencing. We assigned patients to ‘myopathic’ or ‘myoclonic’ EOA subgroups, when myopathy or myoclonus was described in the medical records as major comorbid EOA pathology *and* when myopathic or myoclonic features are phenotypically described in the OMIM database^1^. The ‘myopathic’ co-morbidity subgroup (EOA_MY OPATHIC_) involved 11 patients with FA (*n* = 8); KSS (*n* = 1); MHBD (*n* = 1); and NARP (*n* = 1) gene-mutations. The ‘myoclonic’ co-morbidity subgroup (EOA_MY OCLONIC_) involved four GOSR2 patients with spontaneous, multifocal myoclonus and action-induced enhancement, at the upper extremities, face and lower extremities (van Egmond et al., 2014). In all four EOA_MY OCLONIC_ patients, the medical records described clinical presence of comorbid myoclonus, which was also assessable during videotaped motor task performances (in 3 of 4 patients by 2 of 3 observers and in 1 patient by 1 of 3 observers). The remaining ‘other’ subgroup involved 13 patients, with neither ‘myopathic’ nor ‘myoclonic’ co-morbidity. In all patients, we reported the presence of secondary movement disorder features when at least 2 of 3 independent observers had assessed the same secondary feature, in accordance with the clinical phenotype. For patient characteristics, see **Table 1**.

**Table 1:** Patient characteristics.

	Age	EOA onset	EOA duration^#^	Ambulant *n* (%)	2^nd^MD features video 2/3 obs; *n* (%)	Disease co-morbidity	Medicationˆ
Total group (*n* = 28)^$^	15.5 (6–34)	3 (0–11)	11 (3–25)	19 (68)			
EOA_MY OPATHIC_ (*n* = 11)^$^	17 (8–27)^ns^	4 (1–11)	7 (3–25)^ns^	4 (36)		Hypertr cardiomyo (*n* = 6) Tachycardia (*n* = 2) Scoliosis (*n* = 2) Insulin deficiency (*n* = 1) AV-block (*n* = 1) Hypoparathyroidism (*n* = 1)	Idebenone (*n* = 5) Amiodaron (*n* = 1) Baclofen (*n* = 1) Magnesium (*n* = 1) Carbamazepine (*n* = 1)
EOA_MY OCL_ (*n* = 4)	15 (6–25)^ns^	3 (1–3)	13 (3–22)^ns^	4 (100)	3 (75) Myoclonus	Refractory epilepsy (*n* = 3)	Valproic acid (*n* = 2) Levetiracetam (*n* = 2) Clonazepam (*n* = 3) Clobazam (*n* = 1) Topiramate (*n* = 1)
EOA_OTHER_ (*n* = 13)	15 (8–34)	2 (0–11)	13.5 (8–23)	11 (85)	2 (15) Dystonia 2 (15) Chorea	IgA-deficiency (*n* = 1)	Miglustat (*n* = 1) Sultiame (*n* = 1) Levetiracetam (*n* = 2) Valproaic acid (*n* = 1) Clonazepam (*n* = 1) Dipiperon (*n* = 1) Melatonin (*n* = 1) Concerta (*n* = 1)
EOA_NON-MOY P_ (*n* = 17)	15 (6–34)^ns^	2 (0–11)	13.5 (3–23)^ns^	15 (88)			
EOA_NON-MY OCL_ (*n* = 24)	15.5 (8–34)^ns^	3 (0–11)	11 (3–25)^ns^	15 (63)			

*EOA, early onset ataxia; EOA onset and duration: median value (range); # = scores are normally distributed; ambulant: number (%) ambulant patients; 2^*nd*^MD features video 2/3 obs = number (%) of secondary movement disorder features recognized by all 2 of the 3 observes; Medicationˆ = medication with published side effects on motor function; Hypertr cardiomyo, hypertrophic cardiomyopathy; $ = data about disease onset and disease duration missing in 1 patient; EOA_*MYOPATHIC*_, EOA with reported comorbid myopathy; EOA_*NON-MYOP*_, EOA and absent comorbid myopathy (EOA_*MYOCLONUS*_ + EOA_*OTHER*_); EOA_*MYOCL*_, EOA with comorbid myoclonus; EOA_*NON-MYOCL*_, EOA and absent comorbid myoclonus (EOA_*MYOPATHIC*_ + EOA_*OTHER*_); ns, age and disease duration did not significantly differ between EOA_*MYOPATHIC*_ and EOA_*NON-MYOP*_ and between EOA_*MYOCL*_ and EOA_*NON-MYOCL*_ (Mann–Whitney *U*; Student’s *t*-test).*

### Assessments

In pediatric EOA patients, we investigated the SARA_GAIT/POSTURE_ construct validity by determining the: (1) inter-observer reliability, (2) convergent validity, and (3) discriminant validity.

#### Inter-Observer Reliability

For the inter-observer reliability, we determined the Interclass Correlation Coefficient (ICC) of the SARA_GAIT/POSTURE_ video-ratings by three independent pediatric neurologists, according to the official SARA guidelines (Schmitz-Hubsch et al., 2006).

#### Convergent Validity

For convergent validity, we correlated SARA_GAIT/POSTURE_ [i.e., summed gait, stance, and sitting sub-scale scores (Schmitz-Hubsch et al., 2006] with other rating scale scores for coordinated motor function, including ASMK [dynamic balance (Klockgether et al., 1998)]; PBS [static balance (Franjoine et al., 2010)]; GMFCS-E&R (Palisano et al., 1997, 2008), Dutch version^2^; SARA_KINETIC_ (kinetic function of upper and lower limbs) (Schmitz-Hubsch et al., 2006); AS (kinetic function of the upper limbs (Trouillas et al., 1997) and, finally also SARA_TOTAL_ [summed ataxia scores in gait/posture, kinetic, and speech domains (Schmitz-Hubsch et al., 2006)]. To prevent unnecessary test burden and exhaustion of the patient, we planned investigations during successive hospital visits for clinical reasons. For latent time intervals between tests, see Supplementary Table I.

For information about SARA, AMSK, PBS, GMFCS-E&R, and AS testing, see Appendix B. The ASMK (Klockgether et al., 1998) and GMFCS (Palisano et al., 2008) data were compiled from patient records and interviews. The PBS (Franjoine et al., 2010) scores were provided by one independent investigator, blinded for the results of the other test scores. In children, the reliability of this method was shown to be very high (ICC.997) (Franjoine et al., 2003).

#### Discriminant Validity

For discriminant validity, we determined the potentially confounding influence by comorbid EOA factors, consisting of (1) myopathic muscle weakness and (2) myoclonus on the SARA_GAIT/POSTURE_ scores. We assessed MF by hand held dynamometry (CITEC; C.I.T. Technics, Haren, Groningen, Netherlands) (Beenakker et al., 2001). We determined summed total muscle force (MF_TOTAL_), upper extremity muscle force (MF_UE_), lower extremity muscle force (MF_LE_), and proximal muscle force (MF_PROX_). For detailed information of the tested muscles per item, see Appendix B. As the normality of pediatric MF depends on age, weight and sex, we expressed outcomes as Z-scores from the corrected normal values (Beenakker, 2005).

As ‘ataxia’ and/or ‘myoclonus’ could theoretically prohibit accurate muscle activation and/or MF assessment, we controlled whether paretic measurements (Z-scores < -2 SD) were consistent with MU abnormalities of the same muscles. MU images (of the biceps, rectus femoris, and tibial anterior muscles) were obtained in accordance with a standard protocol and settings (Sival et al., 2011; Brandsma et al., 2012). Two MU experts independently classified MU images as: ‘myopathic,’ ‘neuropathic,’ ‘combined’ (i.e., myopathic and neuropathic) or ‘none’ (in absence of myopathic or neuropathic abnormalities). In a previous publication, we have shown the reliability of this method (Brandsma et al., 2014b). Myopathic abnormalities are characterized by homogeneously increased MU density and/or muscle atrophy in a proximal to distal distribution. Neurogenic muscle abnormalities are characterized by MU inhomogeneity.

### Correlations and Comparisons

For assessment of convergent validity, we correlated SARA_GAIT/POSTURE_ (Schmitz-Hubsch et al., 2006) with the scores from: ASMK (dynamic balance), PBS (static balance), GMFCS-E&R, AS, SARA_KINETIC_, and SARA_TOTAL_. For the assessment of discriminant validity, we correlated SARA_GAIT/POSTURE_ sub-scale scores with MF Z-scores. The correlations between SARA_GAIT/POSTURE_ scores and MF Z-scores were subsequently stratified for EOA subgroups with and without comorbid myopathy. To evaluate the potential influence by myopathy and myoclonus on the SARA_GAIT/POSTURE_ scores, we calculated the relative contribution of SARA_GAIT/POSTURE_ to the total SARA scores (i.e., SARA_GAIT/POSTURE_ %sub-score = [median gait score/median total score] × 100%), and we compared outcomes between myopathic versus non-myopathic and myoclonic versus non-myoclonic subgroups. For further insight, we also compared the SARA_KINETIC_ sub-score percentages (i.e., SARA_KINETIC_ %sub-score = [median kinetic score/median total score] × 100%) between all subgroups.

### Statistical Analysis

We performed statistical analysis using SPSS statistics 22.0. We determined normality of age, time differences between assessments, median SARA scores, ASMK scores, PBS scores, GFMCS-E&R scores, AS scores and MF z-scores both graphically and by the Shapiro–Wilk test. Correlation results were interpreted by the Evans criteria [<0.20 very weak; 0.2 to 0.39 weak; 0.40 to 0.59 moderate; 0.6 to 0.79 strong, and 0.8 to 1 as very strong (Evans, 1996)]. All statistical tests were two-sided. *p*-values <0.05 were considered as statistically significant. We applied the Bonferroni correction to adjust the *p*-value for multiple comparisons on the same data.

## Results

### Scale Descriptives and Inter-Observer Agreement

For descriptives of SARA, ASMK, PBS, GMFCS-E&R, and MF scores, see **Table 2**. The included patients revealed a binary distribution of ASMK scores (ASMK scores 1 and 3), corresponding with ambulant and non-ambulant function, respectively. There was no association between cross-sectional SARA scores and age or disease duration (Spearman’s Rho, *r*_s_ = 0.110; *p* = 0.58; and *r*_s_ = -0.108; *p* = 0.59, respectively). For missing data, see Appendix A. The inter-observer agreement (ICC) of SARA_GAIT/POSTURE_, SARA_TOTAL_ and SARA_KINETIC_ was high (0.97; 0.97; and 0.88, respectively).

**Table 2:** Rating scale scores per EOA group.

	Total group (*n* = 28)	EOA_MY OPATHIC_ (*n* = 11)	EOA_NON-MY OP_ (*n* = 17)	*p*-value	EOA_MY OCL_ (*n* = 4)	EOA_NON-MY OCL_ (*n* = 24)	*p*-value
**SARA scores**							
Total							
Median (p25–p75)	14.5 (9.1–25.6)	27 (14.8–30.5)	11 (8.5–18)	0.022^∗^	13.5 (10.1–18.8)	15.1 (8.7–27.8)	0.694
Min–max	5–34.5	5.3–34.5	5–29.8		9–20.5	5–34.5	
**Gait/posture**							
Median (p25–p75)	6 (4–14.5)	15 (5–18)	5 (3.3–6.5)	0.004^∗∗^	5 (3.3–6.8)	6 (4–15)	0.306
Min–max	3–18	4–18	3–15		3–7	3–18	
**Kinetic#**							
Median (p25–p75)	5.3 (3.6–9.2)	8 (4.3–10)	5 (3.3–8)	0.144	6 (4.6–10.4)	5.3 (3.5–9.2)	0.469
Min–max	1.5–11.5	1.5–10.5	1.5–11.5		4.5–11.5	1.5–10.5	
**ASMK scores**							
Median (p25–p75)	1 (1–3)	3 (1–3)	1 (1–1)	0.009^∗∗^	1 (1–1)	1 (1–3)	0.117
Min–max	1–3	1–3	1–3		1–1	1–3	
**PBS scores**							
Median (p25–p75)	42 (4–50)	3.5 (0–43.1)	45 (25.3–50.4)	0.005^∗∗^	43.8 (34.6–48.8)	32.3 (3.8–50)	0.476
Min–max	0–55	0–50	4–55		32–50	0–55	
**GMFCS-E&R**							
Median (p25–p75)	1 (1–3)	4 (2–4)	1 (1–2)	0.000^∗∗^	1,5 (1–2)	2 (1–4)	0.243
Min–max	1–5	2–5	1–4		1–2	1–5	
**Archimedes spiral**							
Median (p25–p75)	1.5 (1–2.9)	2 (0.8–3)	1 (1–2.9)	0.606	2.3 (1.3–3.6)	1 (1–2.8)	0.279
Min–max	0–4	0–4	0–4		1–4	0–4	
**MF (z-scores)**							
Median (p25–p75)	-1.2 (-3.5 to -0.4)	-3.2 (-4.8 to -1.3)	-0.6 (-1.3 to -0.2)	0.004^∗∗^	-0.6 (-1.9 to -0.1)	-1.3 (-4.2 to -0.5)	0.245
Min–max	-5.9 to 0.4	-5.9 to -0.7	-4.5 to 0.4		-2.2 to -0.1	-5.9 to 0.4	

*SARA_TOTAL_, total score of the Scale for Assessment and Rating of Ataxia; ASMK, Ataxia Severity Measurement according to Klockgether; PBS, Pediatric Balance Scale; GMFCS-E&R, Gross Motor Function Classification Scale – extended and revised version; MF, total muscle force; EOA_MYOPATHIC_, EOA with reported comorbid myopathy; EOA_NON-MYOP_, EOA with absent comorbid myopathy (EOA_MYOCLONUS_ + EOA_OTHER_); EOA_MYOCL_, EOA with reported comorbid myoclonus; EOA_NON-MYOCL_, EOA with absent myoclonus (EOA_MYOPATHIC_ + EOA_OTHER_); p25–p75, lower and upper quartile; min, minimum; max, maximum; # = scores are normally distributed; p-values ^∗^p < 0.05, ^∗∗^p < 0.01 (Mann–Whitney U-test). The EOA_MYOPATHIC_ subgroup reveals higher SARA_TOTAL_, SARA_GAIT/POSTURE_ and ASMK scores and lower PBS and muscle force scores than EOA_NON-MYOP_. The EOA_MYOCL_ and EOA_NON-MYOCL_ subgroups did not significantly differ.*

### Convergent Validity: The Association between SARA Scores, Ataxia Severity Measurement Scale (ASMK), Balance Performance (PBS), Gross Motor Functional Classification Scale (GMFCS-E&R), and Archimedes Spiral (AS)

**FIGURE 1:**
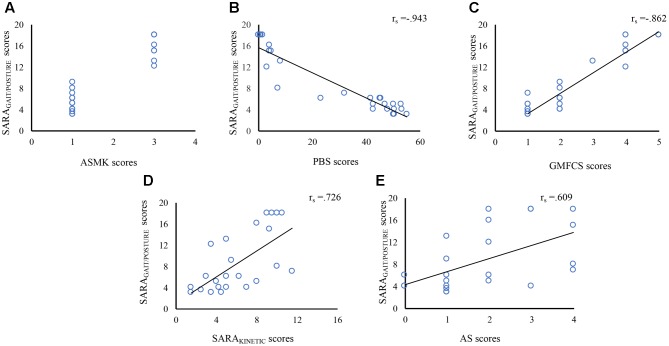
Correlation between SARA_GAIT/POSTURE_ sub-scores and ASMK, PBS scores, GMFCS-E&R, SARA_KINETIC_, and AS. The x-axis indicates ASMK scores **(A)**, PBS scores **(B)**, GMFCS-E&R classification **(C)**, SARA_KINETIC_ scores **(D)**, AS scores **(E)**. The y-axis indicates the SARA_GAIT/POSTURE_ scores **(A–E)**. SARA_GAIT/POSTURE_ scores were associated with ASMK, PBS scores, GMFCS-E&R, SARA_KINETIC_, and AS scores. SARA, Scale for Assessment and Rating of Ataxia; ASMK, Ataxia Severity Measurement according to Klockgether; PBS, Pediatric Balance Scale; GMFCS-E&R, Gross Motor Function Classification Scale-the extended and revised version; AS, Archimedes Spiral.

SARA_GAIT/POSTURE_ and SARA_TOTAL_ scores were (very) strongly associated with ASMK, PBS, GMFCS-E&R, SARA_KINETIC_, and AS scores; see **Table 3** and **Figure 1**. For comparison of SARA scores between the ambulant subgroup (AMSK score 1) and the non-ambulant subgroup (AMSK score 3), see Supplementary Table II. SARA_GAIT/POSTURE_ sub-analysis for active balance (SARA_WALKING_) and passive balance (SARA_STANCE_/_SITTING_) revealed high correlations: (1) between SARA_WALKING_ items and ASMK scores, and (2) between SARA_STANCE_/_SITTING_ and PBS scores (Spearman’s Rho: *r*_s_ = 0.867 and *r*_s_ = 0.917, respectively; *p* < 0.001). SARA_GAIT/POSTURE_ was also correlated with SARA_KINETIC_ (kinetic function of the upper *and* lower limbs; *r*_s_ = 0.726; *p* < 0.001) and with AS (kinetic function of the upper limbs; *r*_s_ = 0.609; *p* = 0.002). See **Table 3** and **Figure 1**.

**Table 3:** Correlations between SARA scores and other measurements of coordination.

	SARA_GAIT/POSTURE_	SARA_TOTAL_	ASMK	PBS	GMFCS-E&R	SARA_KINETIC_^#^	AS
SARA_GAIT/POSTURE_	-	0.935ˆ*	0.815ˆ*	-0.943ˆ*	-0.862ˆ*	0.726ˆ*	0.609ˆ*
SARA_TOTAL_	0.935ˆ*	-	0.772ˆ*	-0.911ˆ*	0.767ˆ*	0.887ˆ*	0.805ˆ*
ASMK	0.815ˆ*	0.772ˆ*	-	-0.817ˆ*	0.848ˆ*	0.474	0.489
PBS	-0.943ˆ*	-0.911ˆ*	-0.817ˆ*	-	-0.870ˆ*	-0.685ˆ*	-0.640ˆ*
GMFCS-E&R	-0.862ˆ*	0.767ˆ*	0.848ˆ*	-0.870ˆ*	-	0.510	0.461
SARA_KINETIC_^#^	0.726ˆ*	0.887ˆ*	0.474	-0.685ˆ*	0.510	-	0.846ˆ*
AS	0.609ˆ*	0.805ˆ*	0.489	-0.640ˆ*	0.461	0.846ˆ*	-

*SARA_TOTAL_, total score of the Scale for Assessment and Rating of Ataxia; SARA_GAIT/POSTURE_, SARA gait and posture sub-scales; ASMK, Ataxia Severity Measurement according to Klockgether; PBS, Pediatric Balance Scale; GMFCS-E&R, Gross Motor Function Classification Scale – extended and revised version; AS, Archimedes Spiral; # = Scores are normally distributed; values represent Spearmans Rho; ^∗^correlations are considered statistically significant with p ≤ 0.002 (Bonferroni correction for 21 comparisons). SARA_GAIT/POSTURE_ and SARA_TOTAL_ correlated strongly with other parameters for coordination measurement.*

### Discriminant Validity

(a) Association between SARA scores and muscle force

**FIGURE 2:**
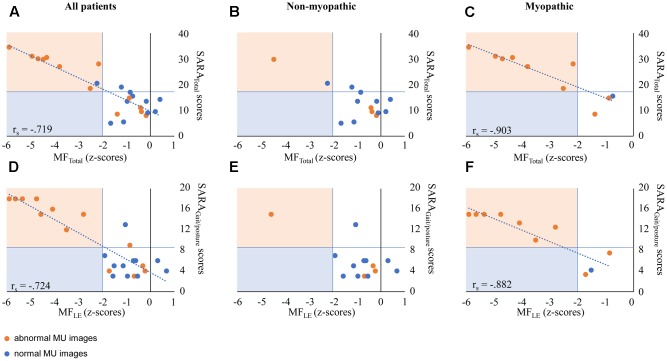
Correlation between SARA scores and MF in EOA patients. **(A,D)** Represent outcome data in all patients. **(B,E)** Represent outcome data in non-myopathic patients. **(C,F)** Represent outcome data in myopathic patients. Orange markers represent patients with abnormal MU characteristics (by expert opinion). **(A–C)** The x-axis indicates MF_TOTAL_ z-scores; the y-axis indicates SARA_TOTAL_ scores. **(D–F)** The x-axis indicates MF_LE_ z-scores; the y-axis indicates SARA_GAIT/POSTURE_ scores. *r*_s_ values are presented in case of significant correlations. SARA, Scale for Assessment and Rating of Ataxia; MF, muscle force; LE, lower extremities. In heterogeneous EOA patients, the association between SARA scores and MF is attributed to outcomes of myopathic patients.

In the *total EOA group*, SARA_GAIT/POSTURE_ and SARA_TOTAL_ revealed strong correlations with muscle weakness of the lower extremities (MF_LE_) and proximal muscles (MF_PROX)_ (MF_LE_ and MF_PROX_). In the ‘myopathic’ subgroup, SARA_GAIT/POSTURE_ and SARA_TOTAL_ revealed very strong correlations with muscle weakness of the lower extremities. For all *r*-values, see **Table 4** and **Figure 2**. In the myopathic subgroup, we controlled whether dynamometry and MU assessments corresponded with myopathic pathology (see **Table 5**). MU analysis revealed pure myopathic changes in 60% and combined myopathic/neurogenic changes in 30%. In the non-myopathic subgroup, the above mentioned correlations with muscle weakness were absent. This group revealed one child with neuropathic alterations and substantial muscle weakness, revealing a similar association between SARA_GAIT/POSTURE_ scores and muscle weakness as the myopathic group. For subgroup correlations, see **Table 4** and **Figures 2A–F**.

**Table 4:** Correlations between SARA scores and muscle force.

	Total group	EOA_MY OPATHIC_	EOA_NON-MY OP_
	*r*-values	*r*-values	*r*-values
SARA_Total_-MF_Total_^∧^	-0.719^∗∗^	-0.903^∗∗^	-0.308
SARA_GAIT/POSTURE_-MF_LE_^∧^	-0.724^∗∗^	-0.882^∗^	-0.320
SARA_GAIT/POSTURE_-MF_Prox_^∧^	-0.690^∗∗^	-0.894^∗∗^	-0.248
SARA_KINETIC_^#^-MF_UE_^#^	-0.574^∗^	-0.619	-0.410
SARA_KINETIC_^#^ -MF_Prox_^∧^	-0.516	-0.564	-0.293

*EOA_MYOPATHIC_, EOA with reported comorbid myopathy; EOA_NON-MYOP_, EOA with absent comorbid myopathy (EOA_MYOCLONUS_ + EOA_OTHER_); MF, muscle force; LE, lower extremities; UE, upper extremities; Prox, proximal muscles; SARA_TOTAL_, total SARA score; SARA_GAIT/POSTURE_, SARA gait sub-score; SARA_KINETIC_, SARA kinetic subscore; # = Scores are normally distributed; ˆ = Spearmans Rho (r-value = r_S_-value); correlations are considered statistically significant with p < 0.01 (Bonferroni correction for five comparisons). ^∗^p < 0.01; ^∗∗^p < 0.001. In the EOA_MYOPATHIC_ subgroup, SARA_TOTAL_ and SARA_GAIT/POSTURE_ scores correlate with MF.*

**Table 5:** Muscle Ultrasound Abnormalities in myopathic and non-myopathic patients.

	EOA_MY OPATHIC_ (*n* = 10)	EOA_NON-MY OP_ (*n* = 14)
Myopathic muscle abnormalities	*n* = 6 (60%)	
Neurogenic muscle abnormalities		*n* = 4 (29%)^∗^
Combined myopathic/neurogenic muscle abnormalities None of the above	*n* = 3 (30%) *n* = 1 (10%)	*n* = 10 (71%)

*EOA_MYOPATHIC_, EOA with reported comorbid myopathy; EOA_NON-MYOP_, EOA with absent comorbid myopathy (EOA_MYOCLONUS_ + EOA_OTHER_); ^∗^corresponding diagnoses were: ataxia telangiectasia (n = 1). Nieman–Pick’s disease (n = 1) and unknown (n = 2).*

(b) Association between SARA scores, myopathy and myoclonus

**FIGURE 3:**
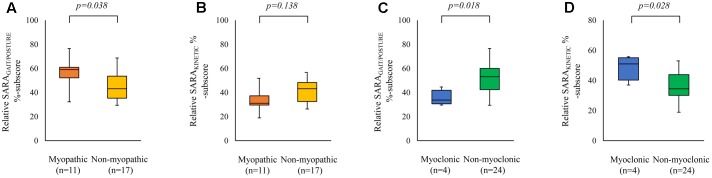
Influence of myopathy and myoclonus on SARA %sub-scores. The x-axis represents the EOA phenotypes (myopathic versus non-myopathic, and myoclonic versus non-myoclonic). The y-axis represents the median SARA_GAIT/POSTURE_ %sub-score (i.e., [SARA_GAIT/POSTURE_ sub-score/median total score] × 100%, **A,C**); and the median SARA_KINETIC_ %sub-score (i.e., [median SARA_KINETIC_ score/median total score] × 100%, **B,D**). Boxes represent lower quartile, median and upper quartile; whiskers represent the minimum and maximum relative %sub-score. SARA_GAIT/POSTURE_, SARA gait and posture sub-score; SARA_KINETIC_, SARA kinetic sub-score. Comparing the %contribution of the SARA_GAIT/POSTURE_ to SARA_TOTAL_ between myopathic versus non-myopathic subgroups, revealed a significantly higher %contribution of the SARA_GAIT/POSTURE_ in the myopathic subgroup **(A)**, whereas the %contribution of the SARA_KINETIC_ was not significantly different between both groups **(B)**. Comparing the %contribution of the SARA_KINETIC_ to SARA_TOTAL_ between myoclonic versus non-myoclonic subgroups, revealed a significantly higher %contribution in the myoclonic subgroup **(C)**, whereas the %contribution of the SARA_GAIT/POSTURE_ revealed a significantly lower %contribution in the myoclonic subgroup **(D)**.

**FIGURE 4:**
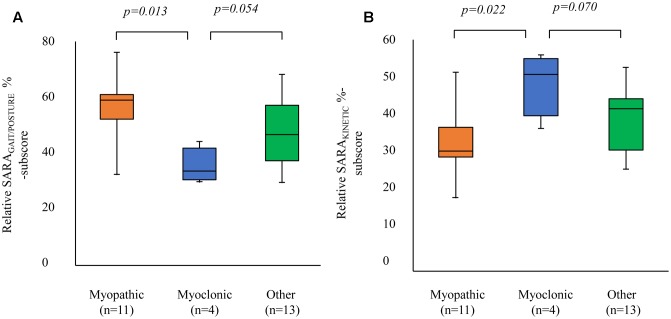
Comparison of relative SARA %sub-scores between co-morbidity subgroups. The x-axis represents the EOA phenotypes [myopathic, myoclonic, and other (non-myopathic and non-myoclonic)]. The y-axis represents: **(A)** the median SARA_GAIT_ %sub-score (i.e., [SARA_GAIT_ score/median total score] × 100%) and **(B)** the median SARA_KINETIC_ %sub-score (i.e., [median SARA_KINETIC_ score/median total score] × 100%). Boxes represent lower quartile, median and upper quartile; whiskers represent the minimum and maximum relative %-sub-score. SARA_GAIT/POSTURE,_ SARA gait and posture sub-score; SARA_KINETIC_, SARA kinetic sub-score. Myoclonic EOA phenotypes reveal a relatively smaller %SARA_GAIT_ than %SARA_KINETIC_ sub-scores compared to the other subgroups.

Comparing EOA subgroups, revealed the highest %contribution of the SARA_GAIT/POSTURE_ to the SARA_TOTAL_ (i.e., SARA_GAIT/POSTURE_/SARA_TOTAL_ × 100%) in the myopathic subgroup (Mann–Whitney *U*, *p* = 0.038), see **Figure 3**. Comparing the %contribution of the SARA_GAIT/POSTURE_ to SARA_TOTAL_ between myoclonic versus non-myoclonic subgroups, revealed a significantly lower %contribution of the SARA_GAIT/POSTURE_ in the myoclonic subgroup (Mann–Whitney *U*, *p* = 0.018, see **Figure 3**). Conversely, we observed the highest %contribution of the SARA_KINETIC_ to SARA_TOTAL_ (i.e., SARA_KINETIC_/SARA_TOTAL_ × 100%) in the myoclonic subgroup (Mann–Whitney *U*, *p* = 0.028), see **Figure 3**. For subgroup comparisons between myoclonic, myopathic, and other (non-myoclonic and non-myopathic), see **Figure 4**.

## Discussion

In children and young adults with EOA, we aimed to investigate the construct validity of SARA_GAIT/POSTURE_ sub-scores. SARA_GAIT/POSTURE_ sub-scores revealed a high inter-observer agreement (ICC) and were strongly associated with other quantitative scales for coordinative motor function, such as: active and static balance (ASMK, PBS), kinetic limb performances (SARA_KINETIC_, AS) and total ataxia scores (SARA_TOTAL_). Furthermore, we also observed a strong correlation between SARA_GAIT/POSTURE_ sub-scores and the classification levels of the GMFCS (E&R), which is originally designed for the assessment of functional motility in children with cerebral palsy (Palisano et al., 1997, 2008). The discriminant validity of the SARA_GAIT/POSTURE_ subscale between the measurement of ataxia and co-morbidity factors (muscle weakness and myoclonus) was incomplete. In children and young adults with EOA, we conclude that SARA_GAIT/POSTURE_ scores are reliable. However, SARA_GAIT/POSTURE_ parameters discriminate insufficiently between the influence by ataxia and muscle weakness. This implicates that gait and posture scores should be interpreted in homogeneous EOA subgroups that take comorbid muscle weakness into account.

In previous EOA studies, we have shown that tools for the assessment of ataxic gait may contribute to the early recognition of indisputable EOA in young patients (Lawerman et al., 2016). Furthermore, well-validated clinical biomarkers for EOA gait and posture assessment are useful for the evaluation of pediatric treatment strategies, targeting at the training of core-muscle function (van Diest et al., 2016; Schatton et al., 2017). In the present study, we observed an excellent inter-observer agreement (ICC) on SARA_GAIT/POSTURE_ sub-scores, which was in the same range as SARA_TOTAL_ and SARA_KINETIC_ sub-scores. These SARA_TOTAL_ outcomes are in agreement with previously published ICC data in adult patients with predominantly AOA phenotypes (Schmitz-Hubsch et al., 2006).

We determined convergent validity of SARA_GAIT/POSTURE_ sub-scores under the premise that all ataxic gait parameters for walking, standing, and balancing would depend on the same integrated cerebellar processing of sensory, visual, and vestibular signals (Takakusaki, 2017) with upper- and lower- limb and trunk motor performances (Sival, 2012; Delabasita et al., 2016). We thus hypothesized that the construct validity of SARA_GAIT/POSTURE_ could be reflected by the association with other coordinative motor function tests requiring cerebellar integration of multimodal signals. Accordingly, we observed that SARA_GAIT/POSTURE_ sub-scores were strongly associated with the tested parameters for coordinated motor function. The SARA_GAIT/POSTURE_ items for active and passive balance were strongly related with ASMK and PBS scores and also with GFMCS classifications, implicating that the closely associated test objectives have a functional significance. Furthermore, SARA_GAIT/POSTURE_ scores were also correlated with kinetic functions of the upper and lower extremities, which can be understood by the fact that gait kinetics (including arm swing, turning, balance and tandem -stance and -gait performances) also require accurate limb kinetics. Finally, SARA_GAIT/POSTURE_ scores appeared strongly associated with SARA_TOTAL_ scores. Although correlated, SARA_GAIT/POSTURE_ and AS scores revealed the lowest correlation. In perspective of the differences in tested cerebellar domains (vermis versus hemispheres) and the differences regarding motor function tasks (gross versus fine motor function tasks), the lower correlation is in accordance with our expectations. As focal cerebellar damage was excluded from the present study group inclusion, one could attribute the above mentioned correlations between different cerebellar domains and/or motor function tasks to global functional pathology of the cerebellum. In young, ataxic EOA patients without focal cerebellar lesions, these results may thus implicate that SARA_GAIT/POSTURE_ scores can provide a global impression of the total ataxia-severity. When ambulant EOA children without focal lesions are too young (<4 years of age) or lack the motivation and/or concentration to complete all SARA motor task performances, SARA_GAIT/POSTURE_ parameters could theoretically provide a fast and easy biomarker to estimate ataxia-progression. Altogether, in children and young adults with distinct EOA features, SARA_GAIT/POSTURE_ can reliably measure ‘ataxic’ gait severity and may also provide a global impression of the total ataxia severity.

We obtained the above mentioned results under the premise that SARA and other coordination scales measure the same objective. However, as already stated for the AS, this is not necessarily correct, as the other biomarkers (such as for active and passive balance, and kinetic function) may measure more than the objective ‘ataxia,’ alone. This implicates that other factors than ataxia could theoretically influence SARA_GAIT/POSTURE_ scores. For instance, in previous studies, we have shown that the age of the child (i.e., cerebellar maturation) has an influence on SARA scores (Sival and Brunt, 2009; Brandsma et al., 2014a). Although mean age-related effects are comparatively small in relation to pathologic SARA scores in ataxic patients, the Childhood Ataxia and Cerebellar Group of the European Pediatric Neurology Society has recently shown that children younger than 8 years of life can also reveal considerable variation in SARA_TOTAL_ scores, which may affect the interpretation of the longitudinal scores (Lawerman et al., 2017). However, as the variation of SARA_GAIT/POSTURE_ sub-scores in young children appeared much smaller (Lawerman et al., 2017), one could use the SARA_GAIT/POSTURE_ sub-scale as an internal control to discriminate between physiological age-related and ataxia effects on the SARA_TOTAL_ scores. To elucidate the SARA_GAIT/POSTURE_ test construct, we also investigated the potential effects of co-morbidity factors on the SARA_GAIT/POSTURE_ sub-scores. SARA_GAIT/POSTURE_ and SARA_TOTAL_ scores revealed an incomplete discriminant validity between ataxia and comorbid ‘muscle weakness.’ Although this does not automatically implicate a causal relationship, absence of a relationship between muscle weakness and SARA_GAIT/POSTURE_ and SARA_TOTAL_ scores cannot be assumed, either. For instance, when the child has difficulties to raise an arm against gravity, or when the child has just sufficient MF to walk with support, muscle weakness is likely to affect the scores. Furthermore, in case of limiting muscle weakness to execute the SARA rating scale task, maximal scores should be given. In the latter case, ataxia itself has not determined the score, but limiting muscle weakness instead. This implicates that the discriminant validity of SARA _GAIT/POSTURE_ sub-scores between muscle weakness and ataxia is incomplete.

Analyzing the patient inclusion of the myopathic EOA cohort, revealed a majority of patients with FA. This underpins our previously reported study data on the association between muscle weakness and ataxia scores in FA children (Sival et al., 2011). Interestingly, in another FA cohort, this association between SARA scores and muscle weakness was not reported (Bürk et al., 2009). However, in the latter study, MF Z-scores were not available, implicating that exact correlations cannot be made. Furthermore, one should be aware that correlations between muscle weakness and SARA scores would require patient sub-groups with sufficient variety in MF. For example, in homogeneous EOA groups with normal physiological muscle strength, the influence by muscle weakness on SARA scores would not be addressed. Similarly, in homogeneous EOA groups with severely progressed muscle weakness (represented by non-ambulant patients), plateauing SARA scores would also obscure an association with muscle weakness. These results implicate that it is advisable to obtain SARA_GAIT/POSTURE_ scores in homogeneous EOA subgroups and to stratify outcomes for substantial variations in muscle weakness. Finally, we investigated the EOA influence of comorbid myoclonus on SARA_GAIT/POSTURE_ sub-scores. In the comorbid myoclonus subgroup, the percentage (%) contribution of SARA_GAIT/POSTURE_ to SARA_TOTAL_ scores was low compared to non-myoclonus subgroup, reflecting a negative effect. Interestingly, the percentage (%) contribution of SARA_KINETIC_ to SARA_TOTAL_ scores was high in the comorbid myoclonus subgroup, compared to non-myoclonus subgroup, implicating a predominant effect of comorbid myoclonus on SARA_KINETIC_, instead of SARA_GAIT/POSTURE_ scores. As myoclonic jerks in GOSR2 patients may start at the upper extremities and increase during intended kinetic limb movements, these findings are understandable.

We are aware that this study has several limitations. First, the EOA patients fulfilling the requirements for patient inclusion are rare, implicating that the number of patients was limited. However, as the present data are obtained in a specialized movement disorder center over a study period of 5 years (with an inclusion rate of 100%), investigation of a larger patient cohort will not easily be accomplished. Second, we realize that statistically significant correlations do not necessarily implicate causality (Field, 2009). But, as significant correlations between SARA_GAIT/POSTURE_ sub-scores and MF were consistently absent in patients without MF loss, our findings do not reject causality, either. Third, to avoid an unacceptable test burden and exhaustion for the patients, we planned different tests during successive medical visits to our outpatient clinic (see Supplementary Table I). However, as latent time intervals between tests would only exert a negative influence on the correlations, the positive inter-correlations between SARA_GAIT/POSTURE_ and other ataxia biomarkers cannot be attributed to it. Fourth, we cannot exclude that other, yet unexplored confounders may also exist (such as neuropathy, concentration, behavior, and tiredness). Altogether, in the perspective of the presented findings, we conclude that SARA_GAIT/POSTURE_ scores are associated with MF loss. In EOA patients with comorbid myopathy, it appears prudent to interpret SARA_GAIT/POSTURE_ scores for the severity of muscle weakness.

## Conclusion

The inter-observer agreement and convergent validity of SARA_GAIT/POSTURE_ scores in EOA patients are high, implicating the reliability of the scores. Regarding the incomplete discriminant validity of the scores, it is advisable to interpret SARA_GAIT/POSTURE_ scores for comorbid muscle weakness.

## Author Contributions

TL: draft of the manuscript, data acquisition, data analysis, interpretation of data. RB: data acquisition, revising the manuscript for important intellectual content. RV: data acquisition, interpretation of data, revising the manuscript for important intellectual content. JvdH: data acquisition, interpretation of data, revising the manuscript for important intellectual content. RL: data acquisition, revising the manuscript for important intellectual content. HK: data interpretation, drafting, and revising the manuscript for important intellectual content. DS: concept and design of the manuscript, data acquisition, interpretation of data, drafting, revising, and final version of the manuscript. All authors approved the final version and agreed to be accountable for all aspects of the work.

## Conflict of Interest Statement

The authors declare that the research was conducted in the absence of any commercial or financial relationships that could be construed as a potential conflict of interest.

## Footnotes

1 Online Mendelian Inheritance in Man, OMIM. McKusick-Nathans Institute of Genetic Medicine, Johns Hopkins University (Baltimore, MD, United States), 24-12-2016. World Wide Web: http://omim.org/.

2 Nederlandse vertaling 2009 NetChild Network for Childhood Disability Research, Utrecht, Netherlands, 15-10-2017. World Wide Web: https://canchild.ca/system/tenon/assets/attachments/000/000/067/original/GMFCS-ER_Translation-Dutch.pdf.

## Abbreviations

AOA: adult onset ataxia
AS: Archimedes Spiral
ASMK: Ataxia Severity Measurement according to Klockgether
EOA: early onset ataxia (starting before the 25th year of life)
FA: Friedreich’s ataxia
GMFCS-E&R: Gross Motor Function Classification Scale- extended and revised version
ICARS: International Cooperative Ataxia Rating Scale
MF: muscle force
MF_LE_: MF z-score of lower extremities
MF_Prox_: MF z-scores of proximal muscles
MF_TOTAL_: total MF z-score
MF_UE_: MF z-score of upper extremities
MU: muscle ultrasound
PBS: Pediatric Balance Scale
SARA: Scale for Assessment and Rating of Ataxia
SARA_TOTAL_: summed total SARA score
SARA_GAIT/POSTURE_: the summed SARA sub-scores for gait, stance and sitting
SARA_KINETIC_: SARA kinetic sub-score
