# Supplementary material for: Construct Validity and Reliability of the SARA Gait and Posture Sub-scale in Early Onset Ataxia
Source: Front Hum Neurosci. 2017 Dec 13;11:605. doi: 10.3389/fnhum.2017.00605 (PMC5733344; doi:10.3389/fnhum.2017.00605)
Supplement: Supplementary file 1 [file Data_Sheet_1.docx]

*Supplementary Information*

**Construct Validity and Reliability of SARA Gait & Posture Sub-scale in**

**Early Onset Ataxia**

**TF Lawerman MSc, R Brandsma MD, RJ Verbeek MD PhD, JH van der Hoeven MD PhD, RJ Lunsing MD PhD, HPH Kremer MD PhD, DA Sival MD PhD***

**Supplementary Information**:

Appendix A: Inclusion of patients

Appendix B: Rating Scale Information

Supplementary Table I

Supplementary Table II

***Correspondence to:**

D.A. Sival, MD PhD

Department of Pediatrics, Beatrix Children’s Hospital,

University Medical Center Groningen, University of Groningen

PO Box 30.001, 9700RB Groningen, the Netherlands

Email: [d.a.sival@umcg.nl](mailto:d.a.sival@umcg.nl)

Phone: +31-50-3611085 / Fax: +31-50-3611787

**1 Appendix A: Patient inclusion per sub study**

*SARA versus PBS*

We assessed the correlation between SARA and PBS scores in 26/28 patients. One patient (CACNA1A-mutation) did not perform the PBS. We excluded one patient (NARP-mutation) because of severe progression and critical illness.

*SARA versus GMFCS*

We assessed the correlation between SARA and GMFCS scores in all 28 patients.

*SARA versus ASMK*

We assessed the correlation between SARA and ASMK scores in all 28 patients.

*SARA* *versus Archimedes Spiral*

We assessed the correlation between SARA scores and the Archimedes Spiral in 24/28 patients. Four patients did not perform the Archimedes Spiral (CACNA1A, FRDA, Poretti-Bolthauser Syndrome and unknown diagnosis).

*SARA versus Muscle Weakness*

We assessed the correlation between SARA scores and muscle weakness in 24/28 patients. Three patients (CACNA1A-mutation (n=2) and one unknown diagnosis) did not perform muscle force assessment. We excluded one patient (NARP-mutation) because of severe progression and critical illness.

*SARA versus Myoclonus*

We explored differences in relative contribution of the kinetic SARA sub-score to the total SARA score in all 28 patients.

**2. Appendix B: Rating Scale Information**

The *SARA* (Schmitz-Hubsch *et al.*, 2006) is a summed ataxia rating scale consisting of 8 items covering three domains (gait, kinetic and speech). SARA scores are composed as: SARA_TOTAL_= SARA_GAIT_ (0-18 points; gait items concern: walking, standing and sitting) + SARA_KINETIC_ 0-16 points; kinetic items concern: finger to nose, finger chase, fast alternating hand movements and heel-shin slide) + SARA_SPEECH_ 0-6 points. The scores quantify ataxia progression.

The *ASMK* (Klockgether *et al.*, 1998) is an ataxia rating scale for the measurement of walking (dynamic gait) impairment in five stages: 0= no gait difficulties; 1= onset of gait difficulties; 2= loss of independent gait, as defined by permanent use of a walking aid or reliance on a supporting arm; 3= confinement to wheelchair, as defined by permanent use of a wheelchair; 4= death.

The *PBS* (Franjoine *et al.*, 2010) is a balance scale, measuring balance during sitting (1 item) and standing (13 items). Scores range from 0 (most severely affected) to 56 (optimal performance).

The *GFMCS* - E&R (Palisano *et al.*, 2008) is an expanded and revised version of the GMFCS (Palisano *et al.*, 1997), consisting of a functional 5-level classification system for the assessment of functional motility (reflecting gross motor function), designed for- and validated in- children with cerebral palsy. As manifestations of gross motor function are dependent on age, the GMFCS - E&R provides separate descriptions per age band, including < 2; 2 to 4; 4 to 6; 6 to 12 and 12 to 18 year of age. General headings per level indicate: I **=** independent walking, without limitations; II = walking with limitations; III = walking with a hand-held mobility device; IV = self-mobility with limitations; may use powered mobility; level V= transported in a manual wheelchair. Patients were scored by the Dutch translation of the GFMCS-E&R^[[1]](#footnote-1)^.

The *Archimedes spiral* (ICARS sub-scale; Trouillas *et al.*, 1997) concerns a 5 point scale quantifying drawing performances over a pre-drawn line of a spiral (i.e. kinetic function of the upper limbs), varying from 0 (normal, no decomposition of movement), 1 (slight deviations without hypermetric swerve), 2 (line with re-crossings and/or hypermetric swerves) 3 (major disturbance with hypermetria and decomposition) to 4 (drawing completely disorganized or impossible).

We assessed *muscle force (MF)* of the neck flexors, shoulder abductors, elbow extensors, elbow flexors, wrist extensors, three-point grip, hip flexors, hip abductors, knee extensors, knee flexors and foot dorsal flexor muscles. Except for the neck muscles, all muscles were bilaterally tested. We determined summed scores of total muscle force (MF_TOTAL_), upper extremity muscle force (MF_UE_), lower extremity muscle force (MF_LE_) and proximal muscle force (MF_PROX_), including shoulder abductors, elbow extensors/flexors, hip flexors/abductors and knee extensors/flexors (Beenakker, 2005).

**3.**

**Supplementary Table I:** Time intervals between tests.

**Legend:** Time intervals indicate the time in months between the first (reference) and subsequent test. The mean time interval between the tests was 8 months. The SARA, the ataxia severity measurement according to Klockgether and the Archimedes Spiral were performed on the same day (not indicated in the Table). Two patients from the myoclonic sub-group had revealed a prolonged dynamometry time interval (due to personal circumstances). As these patients did not reveal leg muscle weakness (< - 2SD), this had not influenced the results. SARA = Scale for Assessment and Rating of Ataxia, PBS= Pediatric Balance Scale, MF=Muscle Force, MU= muscle ultrasound, p25-p75=lower and upper quartile.

| Time Intervals (months) | Total Group | Myopathic | Myoclonic | Other |
| --- | --- | --- | --- | --- |
| **SARA.PBS**  Median (p25-p75)    **SARA.MF**  Median (p25-p75)    **MF.MU**  Median (p25-p75) | 9.8 (0 -12.1)  10.6 (0-14.7)  0 (0-0) | 0 (0-9.7)  0 (0-4.3)  0 (0-0) | 12.9 (8.6-14.1)  34.1 (12.4-58.1)  8.8 (-39.4-24.9) | 10.6 (2.3-12)  11.3 (9.4-13.5)  0 (0-0.6) |

**4. Supplementary Table II:** SARA scores and muscle force in ambulant and non-ambulant patients

**Legend:** Group comparison of SARA scores between ambulant and non-ambulant patients Correlation coefficient (Spearman’s Rho) between SARA scores and muscle force in ambulant and non-ambulant patients. SARA_TOTAL_= total SARA score; SARA_GAIT/POSTURE_ = SARA gait sub-score; MF=muscle force; LE= lower extremities; Prox = proximal muscles; * p<.05; **p<.01; ***p<.001 (Mann-Whitney U test); n.a.= not applicable; ^#^ ambulant and
non-ambulant patients revealed a binary AMSK-score distribution of 1 and 3, respectively.

|  | ambulant (n=19)^#^ | non-ambulant (n=9)^#^ | p-value |
| --- | --- | --- | --- |
| Group comparisons between SARA scores | | | |
| SARA_TOTAL_  Median (p25-p75)  Min-Max | 9.5 (8-14.8)  5-21.5 | 29.8 (23-30.8)  18.5-34.5 | <.001*** |
| SARA_GAIT/POSTURE_  Median (p25-p75)  Min-Max | 4 (3.5-6)  3-9 | 16 (14-18)  12-18 | .014* |
| Correlation between SARA and Muscle Force | | | |
| SARA_Total_-MF_Total_ | -.867** | .013 | n.a. |
| SARA_GAIT/POSTURE_-MF_LE_ | -.796 * | -.056 | n.a. |
| SARA_GAIT/POSTURE_-MF_Prox_^^^ | -.516 | -.011 | n.a. |

1. Nederlandse vertaling © 2009 NetChild Network for Childhood Disability Research, Utrecht, the Netherlands, 15-10-2017. World Wide Web URL: https://canchild.ca/system/tenon/assets/attachments/000/000/067/original/GMFCS-ER_Translation-Dutch.pdf [↑](#footnote-ref-1)
